# CAREER DEVELOPMENT (K) AWARDS AT NIA: PROGRAMS, PEER REVIEW, AND SETTING YOU UP FOR SUCCESS

**DOI:** 10.1093/geroni/igad104.0660

**Published:** 2023-12-21

**Authors:** Shoshana Kahana, Shoshana Kahana

## Abstract

The National Institute on Aging (NIA), one of the 27 Institutes and Centers (ICs) of the National Institutes of Health (NIH), leads the federal government in conducting and supporting research on aging and the health and well-being of older people. The growing investment in training, and specifically career development awards (K), has resulted in substantial growth at NIA. The purpose of this symposium is to provide an overview of the 1) various types of NIH career development awards and programs; 2) effective strategies for preparing strong career development award applications; and 3) NIA peer review process for career development awards. Providing information to attendees of the various career development awards, as well as the critical factors to understand in the process of the peer review process, can optimize future success.

